# Association of the pretreatment lung immune prognostic index with immune checkpoint inhibitor outcomes in patients with advanced hepatocellular carcinoma

**DOI:** 10.1186/s40001-023-01198-0

**Published:** 2023-07-05

**Authors:** Tao Sun, Yusheng Guo, Bo Sun, Lei Chen, Yanqiao Ren, Licheng Zhu, Lijie Zhang, Yiming Liu, Chuansheng Zheng

**Affiliations:** 1 Department of Radiology, Union Hospital, Tongji Medical College, Huazhong University of Science and Technology, Wuhan, 430022 China; 2 Hubei Province Key Laboratory of Molecular Imaging, Wuhan, China; 3 Department of Interventional Radiology, The Fifth Medical Center of Chinese, PLA General Hospital, Beijing, China

**Keywords:** Immune checkpoint inhibitor, Hepatocellular carcinoma, Biomarker, LIPI, dNLR, LDH

## Abstract

**Objective:**

To evaluate whether the pretreatment Lung Immune Prognostic Index (LIPI) is associated with outcomes in advanced hepatocellular carcinoma (HCC) patients under ICI.

**Methods:**

A two-center retrospective study of patients with HCC treated with immune checkpoint inhibitors (ICIs) between January 2018 and January 2021 was performed. Based on pretreatment derived neutrophils/ (leukocytes minus neutrophils) ratio (dNLR) greater than 3 and a lactate dehydrogenase (LDH) level greater than the normal value, patients were stratified into three groups (good LIPI:0 risk factor, intermediate LIPI: 1 risk factor, and poor LIPI: 2 risk factors). The primary endpoints were overall survival (OS) and progression-free survival (PFS). The second endpoints were disease control rate (DCR) and objective response rate (ORR).

**Results:**

In the pooled cohort (*n* = 224), 80 (35.7%) had a good LIPI (zero factor), 91 (40.6%) had intermediate LIPI (one factor), and 53 (23.7%) had poor LIPI (two factors). The median follow-up was 25.1 months. Median OS was 16.8 months, 12.5 months, and 9.5 months for the good, intermediate, and poor LIPI groups, respectively (*P* < 0.0001). Median PFS was 11.8 months, 7.8 months, and 4.0 months for the good, intermediate, and poor LIPI groups, respectively (*P* < 0.0001). Multivariate analysis indicated that the intermediate LIPI and poor LIPI both were independently associated with OS, PFS, and ORR, DCR (*P* < 0.05), as risk factors.

**Conclusion:**

Pretreatment LIPI was correlated with worse outcomes for ICIs suggesting that LIPI could be promising biomarker for advanced HCC patients under ICIs.

## Introduction

Hepatocellular carcinoma (HCC) remains a prevalent cancer, contributing significantly to cancer-related deaths worldwide [1]. Unfortunately, due to a low rate of the early-stage diagnoses, most HCC patients are not eligible for curative treatments, such as surgery or liver transplantation [2]. Immune checkpoint inhibitors (ICIs), particularly those targeting programmed cell death protein 1 (PD-1), have been approved by the FDA for treating advanced HCC [3, 4]. However, the benefits of PD-1 inhibitors have only been observed in a subset of advanced HCC patients, despite promising data. In patients with advanced HCC, single-agent PD-1 inhibitors such as nivolumab and pembrolizumab have shown objective response rates ranging from 12 to 18% [3, 4]. Therefore, it is crucial to search for prognostic biomarkers and screen the appropriate advanced HCC population for PD-1 inhibitor treatment.

Several studies have explored potential biomarkers for treatment response to ICIs [5–7]. While predictive biomarkers such as programmed death ligand 1 (PD-L1) expression, microsatellite instability (MSI) status, and gut microbiota have been shown to play a role in various tumors, the data on their predictive value in HCC patients receiving ICIs remain controversial [5, 8, 9]. As of now, there is still a lack of a reliable biomarker to identify HCC patients who will benefit from ICIs.

Inflammation is a crucial factor in the development and progression of HCC due to the effect of immune resistance [10]. Biomarkers based on systemic inflammation, such as the neutrophil–lymphocyte ratio (NLR), derived NLR (dNLR) and lactate dehydrogenase (LDH), have been studied to measure inflammatory status in various cancers, including HCC [11]. However, the prognostic and predictive value of circulating inflammatory biomarkers for ICIs in HCC is still unknown. Recently, Mezquita proposed the lung immune prognostic index (LIPI), which combines baseline dNLR and LDH, as a prognostic biomarker for patients with non-small-cell lung cancer (NSCLC) treated with ICIs [12]. The prognostic value of LIPI has also been observed in other cancers like renal cell carcinoma, and melanoma [13].

This study aims to evaluate the prognostic value of LIPI in two-center cohort of patients who underwent immunotherapy for advanced HCC. The study also aims to determine whether LIPI can identify progressors in patients who are undergoing ICIs.

## Methods

### Patients

In our study, we analyzed a cohort of 224 patients with advanced HCC who were treated with PD-1 inhibitor (camrelizumab) between January 2018 and January 2021 in two hospitals. The patients were diagnosed with HCC based on the standard of AASLD, either pathologically or clinically. Baseline clinical data, including complete blood cell counts, LDH, and albumin levels, were collected within 14 days prior to the first camrelizumab treatment. This retrospective study was approved by our hospital's ethics committee (UHCT-IEC-SOP-016-03-01), and written informed consent was waived due to the nature of the retrospective study and in accordance with national legislation and institutional requirements.

Inclusion criteria comprised the following: (A) age of 18 years or older; (B) radiological diagnosed with HCC; (C) patients continuously received at least two rounds of carelizumab treatment; (D) measurable tumor lesions on computed tomography [14] or magnetic resonance imaging (MRI).

Exclusion criteria comprised the following: (A) metastatic liver malignant; (B) received locoregional treatment during camrelizumab.

### Camrelizumab treatment

Camrelizumab was administrated intravenously at a dose of 200 mg every 3 weeks. If patients developed serve adverse events (AEs), camrelizumab was interrupted. Symptomatic treatment such as glucocorticoids or immune-suppressant agents were administered, depending on the severity and the affected organs.

### LIPI and outcome definitions

LIPI scores were defined based on dNLR (neutrophil count/ [white blood cell count—neutrophil count]) greater than 3 and LDH greater than LDH normal value. The groups were classified as follows: good group, 0 risk factor; intermediate group, 1 risk factor; poor group, 2 risk factors. The primary outcomes included overall survival (OS, defined as the time from first camrelizumab treatment to death from any cause) and progression-free survival (PFS, defined as the time from first camlizumab treatment to tumor progression according to imRECIST) [15]. Tumor response was evaluated by contrasted MRI or CT according to the imRECIST. Disease control rate (DCR) was defined as the percentage of patients with a complete or partial response, or stable diseased). Objective response rate (ORR) was defined as the percentage of patients with a complete or partial response).

### Statistical analysis

SPSS 24.0 software (IBM, Armonk, NY, USA) was used to perform statistical analyses. Categorical variables were presented by frequency with percentages and continuous variables were presented as the mean ± standard deviation (SD). Comparisons between patients characteristics were performed *χ*^2^ or Fisher exact test for categorical variables and the unpaired *t* test, or Wilcoxon sign-rank test for continuous variables. OS and PFS were analyzed using the Kaplan–Meier method and log rank test. Univariate logistic regression was conducted to evaluate the association between LIPI and ORR and DCR. Univariate Cox proportional hazards regression model analysis was used to identify risk factors affecting OS and PFS. *P* values < 0.05 (two-tailed) were considered statistically significant.

## Results

### Study population

A total of 224 HCC patients were enrolled with a median follow-up of 25.1 months (95%CI 20.3–30.4) in the study. According to the definition of LIPI, 80 (35.7%), 91 (40.6%), and 53 (23.7%) patients were allocated to the good, intermediate, and poor LIPI groups, respectively. The detailed baseline characteristics are listed in Table 1. The median age of HCC patients treated anti-PD-1 was 52.6 years old. Patients predominantly male (81.3%), Child–Pugh stage A–B (92.0%), and hepatitis B virus infection (89.7%). All patients had an Eastern Cooperative Oncology Group performance status (ECOG PS) score of 0 (67.2%) or 1 (32.2%). Among pooled patients, 112 (50.0%) patients were treated with PD-1monotherapy and 112 (50.0%) were treated with PD-1 inhibitor combined with multitargeted tyrosine kinase inhibitors (Lenvatinib, 66.1%; Sorafenib, 33.9%). There was no significant difference between the three groups in the baseline (*P* > 0.05).

**Table 1:** Baseline characteristics

Characteristics	Total population (*N* = 224)	LIPI 0 Good (*N* = 80,35.7%)	LIPI 1 Intermediate (*N* = 91, 40.6%)	LIPI 2 Poor (*N* = 53, 23.7%)	*P* value
Age (years)	52.6 ± 10.5	53.1 ± 13.0	54.9 ± 8.8	53.3 ± 11.2	0.484
Gender					0.296
Male	182(81.3%)	64(80.0%)	78(85.7%)	40(75.5%)	
Female	42(18.8%)	16(20.0%)	13(14.3%)	13(24.5%)	
ECOG performance status					0.676
0	152(67.2%)	57(71.3%)	61(67.0%)	34(64.2%)	
1	72(32.2%)	23(28.8%)	30(33.0%)	19(35.8%)	
Child–Pugh stage					0.112
A	159(71.0%)	65(81.3%)	58(63.7%)	36(67.9%)	
B	47(21.0%)	12(15.0%)	24(26.4%)	11(20.8%)	
C	18(8.1%)	3(3.8%)	9(9.9%)	6(11.3%)	
HBV infection					0.562
No	23(10.3%)	10(12.5%)	7(7.7%)	6(11.3%)	
Yes	201(89.7%)	70(87.5%)	84(92.3%)	47(88.7%)	
Macrovascular invasion					0.165
No	183(81.7%)	68(85.0%)	69(75.8%)	46(86.8%)	
Yes	41(18.3%)	12(15.0%)	22(24.2%0	7(13.2%)	
AFP (ng/ml)					0.178
< 400	93(42.3%)	32(41.0%)	33(37.1%)	28(52.8%)	
> 400	127(57.7%)	46(59.0%)	56(62.9%)	25(47.2)	
Combined with target therapy					0.781
Yes	112(50.0%)	39(48.8%)	48(52.7%)	25(47.2%)	
No	112(50.0%)	41(51.3%)	43(47.3%)	28(52.8%)	

*ECOG* Eastern Cooperative Oncology Group, *BCLC* Barcelona Clinic Liver Cancer, *AFP* a-fetoprotein, *HBV* hepatitis B virus, *LIPI* lung immune prognostic index

### Association of LIPI with ICI survival outcomes

**Fig. 1:**
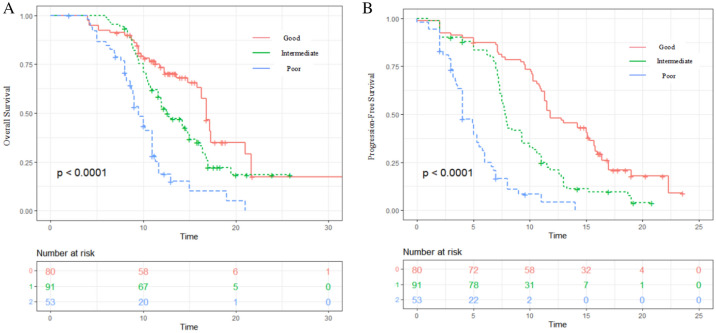
Kaplan–Meier curve for overall survival (OS) (**A**) and progression-free survival (**B**) according to lung immune prognostic index (LIPI). The value of *P* < 0.0001 for both endpoints

LIPI was associated with both OS and PFS (*P* < 0.0001). The median OS for the overall population was 12.7 months (95%CI 10.9 to 14.5) months. Median OS was 16.8 months (95%CI 17.6 to 15.9), 12.5 months (95%CI 10.7 to 14.3), and 9.5 months (95%CI 8.5 to 10.5) for the good, intermediate, and poor LIPI groups, respectively (*P* < 0.001) (Fig. 1A). The one-year OS rates for good, intermediate, and poor LIPI groups were 73.6% (± 5.1%), 52.4% (± 5.3%), and 18.8% (± 6.4%), respectively (*P* < 0.0001). Multivariate analysis showed that intermediate LIPI (HR 2.181; 95%CI 1.416 to 3.361; *P* = 0.001) poor LIPI (HR 4.005; 95%CI 2.467 to 6.501; *P* = 0.001) were associated with a significantly increased risk of death (Table 2).

**Table 2:** Multivariate Cox model analysis for OS and PFS in the population

Variable	OS		PFS	
	HR (95%CI)	*P* value	HR (95%CI)	*P* value
ECOG performance				
0	Ref		Ref	
≥ 1	2.556 (1.784–3.663)	0.021	1.570 (1.163–2.119)	0.003
Child Pugh				
A	Ref		Ref	
B	1.990 (1.385–2.860)	0.043	1.843 (1.334–2.547)	0.072
C	2.339 (1.872–2.409)	0.037	1.992 (1.492–2.672)	0.069
AFP				
≤ 400	Ref		Ref	
> 400	1.252 (1.152–1.309)	0.062	1.679 (1.253–1.998)	0.082
HBV				
No	Ref		Ref	
Yes	1.967 (1.032–2.843)	0.183	1.769 (1.265–2.018)	0.131
Macrovascular Invasion				
No	Ref		Ref	
Yes	1.742 (1.139–2.664)	0.010	2.982 (1.279–4.671)	0.006
Combined with target therapy				
Yes	Ref		Ref	
No	2.493 (2.019–3.052)	0.032	2.835 (2.182–3.461)	0.025
LIPI				
Good	Ref		Ref	
Intermediate	2.181 (1.416–3.361)	0.001	1.872 (1.326–2.642)	0.021
Poor	4.005 (2.467–6.501)	0.001	2.574 (1.717–2.857)	0.008

*OS* overall survival, *PFS* progression-free survival, *HR* hazard ratio, *95%CI* 95% confidence interval, *ECOG* Eastern Cooperative Oncology Group, *BCLC* Barcelona Clinic Liver Cancer, *AFP* a-fetoprotein, *HBV* hepatitis B virus, *LIPI* lung immune prognostic index

Similarly, the median PFS for the overall population was 8.0 months (95% CI 6.9 to 9.1) months. Median PFS was 11.8 months (95%CI 9.5 to 14.1), 7.8 months (95%CI 7.465 to 8.135), and 4.0 months (95%CI 3.2 to 4.8) for the good, intermediate, and poor LIPI groups, respectively (*P* < 0.0001) (Fig. 1B). The one-year PFS rates for good, intermediate, and poor LIPI groups were 48.2% (± 5.6%), 21.1% (± 4.4%), and 8.3% (± 4.3%), respectively (*P* < 0.0001). Multivariate analysis showed that intermediate LIPI (HR 1.872; 95% CI 1.326 to 2.642); *P* = 0.021), poor LIPI (HR 2.574; 95% CI 1.717–2.857; *P* = 0.008) were associated with a significantly increased risk of progression (Table 2).

### Association of LIPI with tumor response under ICI

The relationship of LIPI groups on response outcomes was evaluated in this study. The ORR and DCR in the overall population was 20.1%, and 67.0%, respectively. According to LIPI group, the ORR was 32.5% in the good group,15.4% in the intermediate, and 9.4% in the poor group (*P* = 0.02) (Table 3). The DCR was 91.2% in the good group, 58.3% in the intermediate, and 45.2% in the poor group (*P* = 0.000) (Table 3).

**Table 3:** Relationship between LIPI groups and response to Anti-PD-1 treatment

Response	No. of patients (%)				*P* value
	Overall *n* = 224	LIPI good *n* = 80	LIPI intermediate *n* = 91	LIPI poor *n* = 53	
CR	0 (0)	0 (0)	0 (0)	0 (0)	
PR	45 (20.1%)	26 (32.5%)	14 (15.4%)	5 (9.4%)	
SD	105 (46.9%)	47 (58.7%)	39 (42.9%)	19 (35.85%)	
PD	74 (33.0%)	7 (8.8%)	38 (41.8%)	29 (54.7%)	
ORR	45 (20.1%)	26 (32.5%)	14 (15.4%)	5 (9.4%)	0.002
DCR	150 (67.0%)	73 (91.2%)	53 (58.3%)	24 (45.2%)	0.000

*LIPI* lung immune prognostic index, *CR* complete response, *PR* partial response, *SD* stable disease, *PD* progression disease, *ORR* objective response rate, *DCR* disease control rate

In univariate logistic regression analysis, the intermediate LIPI (OR 2.648; 95% CI 1.267 to 5.534; *P* = 0.010) and poor LIPI (OR 4.622; 95% CI 1.645 to 12.987; *P* = 0.004) were associated with ORR (Table 4). The intermediate LIPI (OR 7.477; 95% CI 5.382 to 9.529; *P* < 0.001) and poor LIPI (OR 12.601; 95% CI 9.183 to 24.305; *P* < 0.001) were associated with DCR (Table 4).

**Table 4:** Univariate logistic regression for response endpoints according to LIPI score

Variable	ORR		DCR	
	OR (95%CI)	*P* value	OR (95%CI)	*P* value
*LIPI*				
Good	Ref.		Ref.	
Intermediate	2.648 (1.267–5.534)	0.010	7.477 (5.382–9.529)	0.000
Poor	4.622 (1.645–12.987)	0.004	12.601 (9.183–24.305)	0.000

*LIPI* lung immune prognostic index, *ORR* objective response rate, *DCR* disease control rate, *OR* odds ratio, *Ref* reference

### Subgroup analysis: association of LIPI with outcomes in PD-1 inhibitor monotherapy or combination therapy

**Fig. 2:**
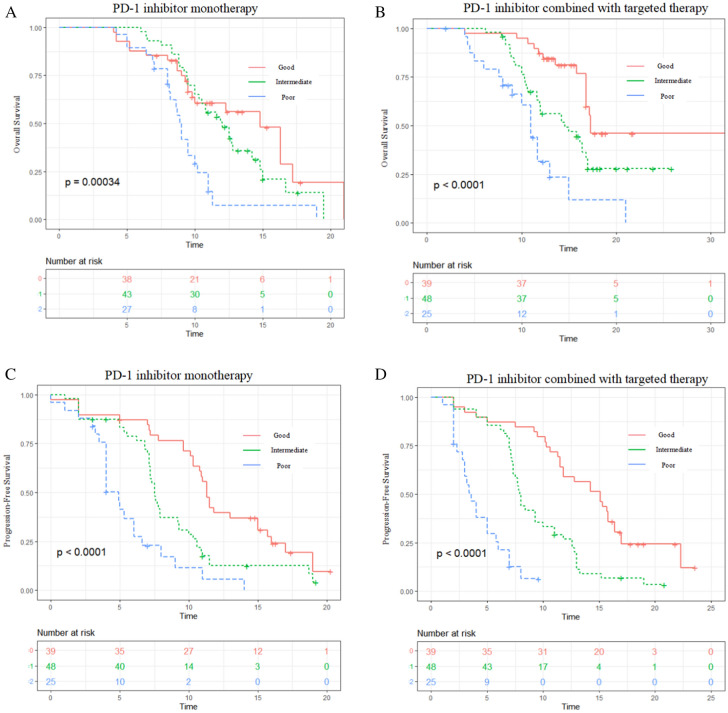
Overall survival (OS) and progression-free survival (PFS) according to subgroup analysis. OS of the PD-1 inhibitor monotherapy cohort (**A**) and of the PD-1 inhibitor combined with targeted therapy cohort (**B**); PFS of the PD-1 inhibitor monotherapy cohort (**C**) and of the PD-1 inhibitor combined with targeted therapy cohort (**D**)

The subgroup analysis was conducted to evaluate the prognostic value of pretreatment LIPI both in PD-1 inhibitor monotherapy and PD-1 inhibitor combined with targeted therapy (Fig. 2). Good LIPI was associated with a significantly longer OS, and PFS compared with intermediate LIPI and poor LIPI, no matter in PD-1 inhibitor monotherapy cohort but also in combination therapy cohort (*P* < 0.001) (Fig. 2A–D).

## Discussion

In this two-center retrospective study, the pretreatment LIPI was firstly used to stratify our HCC population under ICIs into three groups: good LIPI, intermediate LIPI, and poor LIPI. The study included 224 patients who were treated with ICI, median OS and PFS were 12.7 and 8.0 months, respectively. The poor LIPI group was more likely to have progression under ICI and had both shorter PFS (median, 4.0 months) and OS (median, 9.5 months) compared to the intermediate or good LIPI (*P* < 0.001). In subgroup analysis, a significant correlation was found between LIPI and survival outcomes in patients who underwent PD-1 inhibitor monotherapy and PD-1 inhibitor combined with target treatment. The results indicate that LIPI can serve as a prognostic marker for survival/response outcomes in patients with advanced HCC treated with ICI.

Systemic inflammatory status is strongly associated with poor prognosis in various solid tumors [16, 17]. However, the impact of inflammatory status on the benefits of immunotherapy is unclear. Previous studies have shown that some routine blood parameters, such as elevated neutrophils, platelets, hypoalbuminemia, LDH, and dNLR, were associated with poor outcomes in cancer [18, 19]. LDH, with the potential to evaluate tumor burden, is a well-established, independent prognostic factor for survival [20–22]. In their study, Diem et al. found that LDH could serve as a prognostic factor for cancer patients undergoing immunotherapy [23]. Similarly, Proctor et al. evaluated dNLR as a prognostic factor for cancer outcomes in various solid tumors, and found that it had a similar prognostic value to the established NLR [14]. LIPI, which combines LDH and dNLR, has been proposed as a new indicator for predicting the efficacy and prognosis of immunotherapy in patients with different types of cance [24]. In a recent study, Shixue Chen et al. showed for the first time that LIPI is associated with survival and treatment outcomes in HCC patients receiving PD-1 inhibitors [25]. However, subject to small sample, the study stratified patients with HCC into only two groups based on LIPI. Our study divided patients into three groups (good LIPI, intermediate LIPI, and poor LIPI) to better understand the role of LIPI in HCC patients treated with PD-1 inhibitors. Our study assigned HCC patients under PD-1 inhibitor into three groups (good LIPI, intermediate LIPI, and poor LIPI). Benefiting from the above grouping methods, our study not only found that the population of good LIPI had better survival/response outcomes but also found the significant difference in survival/response outcomes between intermediate LIPI group and poor LIPI group.

Additionally, we noted that half of the patients in our study received PD-1 inhibitors in combination with targeted therapy. Our subgroup analysis revealed the population of poor LIPI had worse survival outcomes than those with intermediate or good LIPI in both PD-1 inhibitor monotherapy and combination treatment groups. We know that HCC patients were encouraged to receive immunotherapy combined with targeted therapy based on the results of IMbrave 150 [26]. Therefore, our study, based on real-world data, could provide information for patients with similar conditions for PD-1 inhibitor in clinical practice.

This retrospective study has a few potential limitations. Firstly, some HCC patients were unable to be included due to missing pretreatment clinical data. Secondly, there may be selection bias in the patient population because of the high prevalence of HBV infection in China. Third, although the study included patients from both institutions, the study sample was small. Therefore, further investigations such as large-scale prospective studies are necessary to validate our findings.

## Conclusion

This study is the first to investigate the correlation between the complete pretreatment LIPI score, which includes three groups, and the outcomes of patients with advanced HCC who were treated with ICI. LIPI is a low-cost, simple, and accessible prognostic tool that shows promise for further investigation in large, prospective studies in the context of advanced HCC.

## Data Availability

The data used in the study were available from the correspondence author on reasonable request.

## References

[1] Bray F (2018). Global cancer statistics 2018: GLOBOCAN estimates of incidence and mortality worldwide for 36 cancers in 185 countries. CA Cancer J Clin.

[2] Llovet JM (2016). Hepatocellular carcinoma. Nat Rev Dis Primers.

[3] El-Khoueiry AB (2017). Nivolumab in patients with advanced hepatocellular carcinoma (CheckMate 040): an open-label, non-comparative, phase 1/2 dose escalation and expansion trial. Lancet.

[4] Zhu AX (2018). Pembrolizumab in patients with advanced hepatocellular carcinoma previously treated with sorafenib (KEYNOTE-224): a non-randomised, open-label phase 2 trial. Lancet Oncol.

[5] Goumard C (2017). Low levels of microsatellite instability at simple repeated sequences commonly occur in human hepatocellular carcinoma. Cancer Genomics Proteomics.

[6] Hopkins AM (2017). Predicting response and toxicity to immune checkpoint inhibitors using routinely available blood and clinical markers. Br J Cancer.

[7] Prelaj A (2019). Predictive biomarkers of response for immune checkpoint inhibitors in non-small-cell lung cancer. Eur J Cancer.

[8] Peiffer LB (2022). Composition of gastrointestinal microbiota in association with treatment response in individuals with metastatic castrate resistant prostate cancer progressing on enzalutamide and initiating treatment with anti-PD-1 (pembrolizumab). Neoplasia.

[9] Lin ZF, Qin LX, Chen JH (2022). Biomarkers for response to immunotherapy in hepatobiliary malignancies. Hepatobiliary Pancreat Dis Int.

[10] Hanahan D, Weinberg RA (2011). Hallmarks of cancer: the next generation. Cell.

[11] McMillan DC (2013). The systemic inflammation-based Glasgow Prognostic Score: a decade of experience in patients with cancer. Cancer Treat Rev.

[12] Mezquita L (2018). Association of the lung immune prognostic index with immune checkpoint inhibitor outcomes in patients with advanced non-small cell lung cancer. JAMA Oncol.

[13] Meyers DE (2019). The lung immune prognostic index discriminates survival outcomes in patients with solid tumors treated with immune checkpoint inhibitors. Cancers (Basel).

[14] Proctor MJ (2012). A derived neutrophil to lymphocyte ratio predicts survival in patients with cancer. Br J Cancer.

[15] Hodi FS (2018). Immune-modified response evaluation criteria in solid tumors (imRECIST): refining guidelines to assess the clinical benefit of cancer immunotherapy. J Clin Oncol.

[16] Paramanathan A, Saxena A, Morris DL (2014). A systematic review and meta-analysis on the impact of pre-operative neutrophil lymphocyte ratio on long term outcomes after curative intent resection of solid tumours. Surg Oncol.

[17] Song YJ (2016). Lymphocyte to monocyte ratio is associated with response to first-line platinum-based chemotherapy and prognosis of early-stage non-small cell lung cancer patients. Tumour Biol.

[18] Templeton AJ (2014). Prognostic role of platelet to lymphocyte ratio in solid tumors: a systematic review and meta-analysis. Cancer Epidemiol Biomarkers Prev.

[19] Petrelli F (2015). Prognostic role of lactate dehydrogenase in solid tumors: a systematic review and meta-analysis of 76 studies. Acta Oncol.

[20] Eton O (1998). Prognostic factors for survival of patients treated systemically for disseminated melanoma. J Clin Oncol.

[21] Kelderman S (2014). Lactate dehydrogenase as a selection criterion for ipilimumab treatment in metastatic melanoma. Cancer Immunol Immunother.

[22] Zhao H (2022). Self-supervised learning enables 3D digital subtraction angiography reconstruction from ultra-sparse 2D projection views: a multicenter study. Cell Rep Med.

[23] Diem S (2016). Serum lactate dehydrogenase as an early marker for outcome in patients treated with anti-PD-1 therapy in metastatic melanoma. Br J Cancer.

[24] Benitez JC (2020). The LIPI score and inflammatory biomarkers for selection of patients with solid tumors treated with checkpoint inhibitors. Q J Nucl Med Mol Imaging.

[25] Chen S (2020). Association of the pretreatment lung immune prognostic index with survival outcomes in advanced hepatocellular carcinoma patients treated with PD-1 inhibitors. J Hepatocell Carcinoma.

[26] Finn RS (2020). Atezolizumab plus bevacizumab in unresectable hepatocellular carcinoma. N Engl J Med.

